# Stepwise differentiation and functional characterization of human induced pluripotent stem cell-derived choroidal endothelial cells

**DOI:** 10.1186/s13287-020-01903-4

**Published:** 2020-09-23

**Authors:** Kelly Mulfaul, Joseph C. Giacalone, Andrew P. Voigt, Megan J. Riker, Dalyz Ochoa, Ian C. Han, Edwin M. Stone, Robert F. Mullins, Budd A. Tucker

**Affiliations:** 1 Department of Ophthalmology and Visual Sciences, The University of Iowa Carver College of Medicine, Iowa City, IA 52242 USA; 2 Institute for Vision Research, The University of Iowa, Iowa City, IA 52242 USA

**Keywords:** Induced pluripotent stem cells (iPSCs), Choroid, Choroidal endothelial cells (CECs), Age-related macular degeneration (AMD), Connective tissue growth factor (CTGF)

## Abstract

**Background:**

Endothelial cells (ECs) are essential regulators of the vasculature, lining arteries, veins, and capillary beds. While all ECs share a number of structural and molecular features, heterogeneity exists depending on their resident tissue. ECs lining the choriocapillaris in the human eye are lost early in the pathogenesis of age-related macular degeneration (AMD), a common and devastating form of vision loss. In order to study the mechanisms leading to choroidal endothelial cell (CEC) loss and to develop reagents for repairing the choroid, a reproducible in vitro model, which closely mimic CECs, is needed. While a number of protocols have been published to direct induced pluripotent stem cells (iPSCs) into ECs, the goal of this study was to develop methods to differentiate iPSCs into ECs resembling those found in the human choriocapillaris specifically.

**Methods:**

We transduced human iPSCs with a CDH5p-GFP-ZEO lentiviral vector and selected for transduced iPSCs using blasticidin. We generated embryoid bodies (EBs) from expanded iPSC colonies and transitioned from mTESR™1 to EC media. One day post-EB formation, we induced mesoderm fate commitment via addition of BMP-4, activin A, and FGF-2. On day 5, EBs were adhered to Matrigel-coated plates in EC media containing vascular endothelial cell growth factor (VEGF) and connective tissue growth factor (CTGF) to promote CEC differentiation. On day 14, we selected for CECs using either zeocin resistance or anti-CD31 MACS beads. We expanded CECs post-selection and performed immunocytochemical analysis of CD31, carbonic anhydrase IV (CA4), and RGCC; tube formation assays; and transmission electron microscopy to access vascular function.

**Results:**

We report a detailed protocol whereby we direct iPSC differentiation toward mesoderm and utilize CTGF to specify CECs. The CDH5p-GFP-ZEO lentiviral vector facilitated the selection of iPSC-derived ECs that label with antibodies directed against CD31, CA4, and RGCC; form vascular tubes in vitro; and migrate into empty choroidal vessels. CECs selected using either antibiotic selection or CD31 MACS beads showed similar characteristics, thereby making this protocol easily reproducible with or without lentiviral vectors.

**Conclusion:**

ECs generated following this protocol exhibit functional and biochemical characteristics of CECs. This protocol will be useful for developing in vitro models toward understanding the mechanisms of CEC loss early in AMD.

## Background

Endothelial cells (ECs) line the walls of blood vessels throughout the body, forming the innermost monolayers of vascular tubes. ECs are essential for facilitating the passage of oxygen and vital nutrients from the circulating blood to nourish tissue and organs, while removing CO_2_ and other waste products, thus maintaining tissue homeostasis. Under normal conditions, the EC barrier helps to restrict immune cell infiltration and activation. However, during times of injury, ECs can upregulate secreted and cell surface signals to greatly increase immune cell recruitment and promote an inflammatory response. Importantly, ECs can also aid in tissue repair by triggering blood vessel growth via angiogenesis. Regardless of their location in the body, ECs in any organ system share these basic functions. As such, essentially all ECs express a common set of marker genes including *CD34*, *CD31*, and von Willebrand’s factor (*vWF*) [1]. Depending on their anatomical location, however, there is huge diversity in EC structure and function [2], including the expression of tissue-specific genes. For example, ECs in the arterial system express *NOTCH4* and *EFNB2* whereas ECs in the venous system express *NR2F2* and *EphB4* [3]. Further molecular differences exist between ECs in different organs. Given the variability in endothelial cell subtypes, studying endothelial cells in vitro that are phenotypically similar to those from the relevant tissue is of high interest.

The choroid is a highly specialized vascular network, located between the neural retina and the sclera at the posterior pole of the eye. The choroid develops from the peri-ocular mesenchyme during embryonic development. Fate mapping in mice demonstrates that choroidal endothelial cells are derived from the mesoderm whereas other cell types within the choroid are derived from the neural crest [4]. The choroidal vasculature is essential for supporting healthy vision by providing nutrients to the retinal pigment epithelium (RPE) and photoreceptors while also removing waste products secreted by the RPE. The innermost layer of the choroid closest to the retina is a dense network of large-diameter capillaries, with a lobular arrangement, termed the choriocapillaris. The capillary walls are lined with specialized ECs, which have large fenestrations that allow for diffusion of nutrients, oxygen, and small proteins from the systemic circulation toward the retina, and removal of waste products from the RPE for systemic recycling. The choriocapillaris is supplied by medium-size arterioles that branch off of short posterior ciliary arteries and is drained through a confluence of venules in the vortex vein system near the equator of the eye [5–7].

Loss of choriocapillaris vessels occurs early in the pathogenesis of age-related macular degeneration (AMD). Immunohistochemical and gene expression analyses of human donor eyes demonstrate that endothelial cells lining the choriocapillaris are lost prior to RPE degeneration, creating empty lumens of extracellular matrix termed ghost vessels [8, 9]. Morphometric analysis of the choroidal vasculature in eyes with AMD further supports choriocapillaris vessel loss in early AMD, but similar differences are not readily apparent in the deeper choroidal vessels, suggesting vascular dropout may occur specifically in the capillaries [10]. Standard methods of visualizing choroidal vascular anatomy in vivo include indocyanine green angiography, but this test is invasive, requiring an intravenous injection of dye [11, 12]. Recent advances in clinical imaging have allowed for non-invasive visualization of blood flow in the choroid in vivo and can be used to quantify vessel density. For example, studies using optical coherence tomography angiography (OCTA) have shown reduced vascular density in the choroidal vasculature of AMD patients compared to controls [13]. Most recently, swept-source OCTA, which provides improved visualization of choroidal anatomy, suggests that there are significant decreases in perfusion in the choriocapillaris in early AMD [14] as well as in advanced disease with geographic atrophy [15, 16]. While these in vivo imaging modalities currently do not have the resolution to evaluate endothelial cell loss, the visualized differences in flow could be accounted for by the presence of empty choroidal vessels and are supported by the observation of increased fibrosis and increased ratio of stroma to vessels in AMD eyes by OCTA [17].

Although a number of published protocols have described differentiation of ECs from pluripotent embryonic stem cells [18–21], differences between EC subtypes have revealed the need for refined procedures suitable for directing tissue-specific EC commitment [3, 22]. We have previously shown that connective tissue growth factor (CTGF), when implemented during spontaneous differentiation, plays a key role in driving induced pluripotent stem cells (iPSCs) toward a choroidal endothelial cell (CEC) fate [23]. In the current report, we adopt a directed stepwise differentiation approach using bone morphogenetic protein 4 (BMP-4), fibroblast growth factor 2 (FGF2), activin A, and vascular endothelial growth factor (VEGF) [20], to enrich for mesoderm precursor cells prior to CEC specification using CTGF. This method, which was repeated using iPSCs generated from 3 independent individuals, provides a robust protocol for consistently producing iPSC-derived CECs.

## Methods

**Fig. 1:**
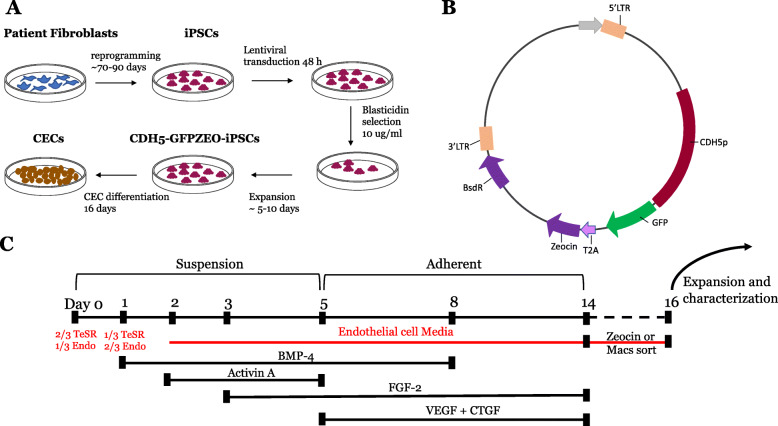
Schematic representation of the stepwise differentiation of iPSC to choroidal endothelial cells. (**a**) Fibroblasts obtained from patient biopsies were reprogrammed to induced pluripotent stem cells (iPSCs). iPSCs were transduced with a lentiviral vector (**b**) expressing the endothelial cell-specific promoter CDH5 driving expression of GFP and zeocin resistance. Forty-eight hours post-transduction, cells were placed under selection with blasticidin to select for positively transduced iPSCs. Following selection, iPSC colonies were expanded prior to stepwise choroidal endothelial cell (CEC) differentiation as outlined in (**c**)

The following protocol describes the stepwise differentiation of iPSCs into choroidal endothelial cells as summarized in Fig. 1. For in-depth, step-by-step instructions, see Additional file 1.

### Lentiviral production

CDH5p-GFP-ZEO lentiviral vector was constructed incrementally. First, the CDH5 promoter was cloned into a pENTR5′-TOPO vector as previously demonstrated [24]. Then, GFP-T2A-Zeocin was amplified from pGreenZEO (System Biosciences) and subcloned into pENTR/D-TOPO. Finally, the pENTR5′CDH5p and pENTR/D-GFP-ZEO were cloned into the pDEST R4R3 Vector II (Thermo Fisher Scientific) using Gateway™ LR Clonase™ II Enzyme mix (Thermo Fisher Scientific). Note that the pDEST R4R3 Vector II includes a blasticidin resistance cassette for selection. The resulting vector was submitted to the Viral Vector Core Facility at the University of Iowa for lentiviral packaging. The CDH5p-GFP-ZEO lentiviral vector is available on Addgene (catalog # 122970). Figure 1b illustrates a simplified plasmid map.

### Viral transduction of iPSCs

Three independent human iPSC lines were maintained in mTeSR™1 (STEMCELL Technologies, USA) base media containing 5× supplement and primocin (100 μg/ml) on Matrigel-coated six-well tissue culture plates. iPSC colonies were incubated with dispase (Thermo Fisher Scientific) at 37 °C for 4–8 min. Dispase was subsequently removed, and iPSC colonies were gently dislodged using mTeSR™1. Cells were subsequently counted and plated at a density of 50,000 cells per well of a 24-well plate. The next day, medium was replaced with mTeSR™1 containing CDH5p-GFP-ZEO lentiviral particles for a final multiplicity of infection (MOI) of 10 (i.e., 10 viral particles per cell). After 48 h, media containing lentiviral particles were replaced with mTeSR™1 containing blasticidin (5 μg/ml). iPSCs were maintained in mTeSR™1 containing blasticidin (5 μg/ml) for 5–7 days, changing medium daily.

### Embryoid body (EB) formation

When iPSC colonies reached 80% confluency, colonies were dissociated using dispase (Thermo Fisher Scientific) at 37 °C for 4–8 min or until the edges of the iPSC colonies began to curl. Dispase was subsequently removed, and iPSCs were gently lifted in DMEM taking care to prevent colonies from breaking up. iPSC colonies were allowed to settle by gravity in a 15-ml conical tube for 5–10 min before resuspension in 6 ml mTESR™1 (STEMCELL Technologies, USA) and 3 ml endothelial cell growth media (R&D systems). iPSCs were transferred to a 60-mm cell culture dish pre-coated with polyHEMA (Sigma-Aldrich). PolyHEMA prevents iPSC colony adhesion. We refer to EB formation as day 0 of differentiation (Fig. 1c).

### EB-based differentiation of iPSC-derived choroidal endothelial cells

On day 1 after EB formation, medium is replaced with 3 ml of mTeSR™1 and 6 ml endothelial cell media supplemented with 20 ng/ml BMP-4 (Miltenyi Biotec). On day 2, media were replaced with 9 ml of endothelial cell media (R&D systems) supplemented with 20 ng/ml BMP-4 (Miltenyi Biotec) and 10 ng/ml activin A (R&D systems). On day 3, EB media were replaced 9 ml of endothelial cell media supplemented with 20 ng/ml BMP-4 (Miltenyi Biotec), 10 ng/ml activin A, and 8 ng/ml FGF-2 (R&D systems). After 2 days (day 5), EBs were collected and adhered, by gravity, to wells of a 6-well cell culture plate pre-coated with Matrigel at a density of 30 EBs per well in endothelial cell media supplemented with 10 ng/ml BMP-4, 8 ng/ml FGF-2, 25 ng/ml VEGFa (Peprotech), and 25 ng/ml CTGF (Peprotech). On day 6, media were replaced with fresh endothelial cell media supplemented with 10 ng/ml BMP-4, 8 ng/ml FGF-2, 25 ng/ml VEGFa, and 25 ng/ml CTGF. After 2 days, on day 8, media were replaced with endothelial cell media supplemented with 8 ng/ml FGF-2, 25 ng/ml VEGFa, and 25 ng/ml CTGF. Cells were maintained in these media from days 8–14, refreshing media every other day. A schematic summary of media changes is presented in Fig. 1c.

### Positive selection for endothelial cell population

A) Zeocin selection

On day 14, differentiating cells were placed under antibiotic selection in endothelial cell media (R&D systems) containing 25 ng/ml of zeocin (Thermo Fisher Scientific) for 48 h.

OR

B) CD31 microbead MACS sort

Alternatively, on day 14, differentiating cells can be positively enriched for CD31-positive endothelial cells using CD31 MACS microbeads. Single cell suspensions were prepared using TrypLE (Thermo Fisher Scientific). Cells were pelleted by centrifugation and resuspended in PBS to a final concentration of 1 × 10^7^ cell/ml. Twenty microliters of FCR blocking agent (Miltenyi Biotech) was added per 60 μl of cells as per the manufacturer’s instructions, followed by 20 μl of CD31 beads (Miltenyi Biotech) per 60 μl of cells. Cells were incubated with the CD31 beads at 4 °C for 15 min with gentle shaking. One milliliter of PBS was added to the cell-bead suspension which was then pelleted by centrifugation. The pellet was resuspended in 1 ml of PBS containing 0.04% non-acetylated BSA. Cells were sorted on an autoMACS sorter using the selection program “possel.” The positive fraction was plated into one well of a 6-well plate pre-coated with Matrigel and maintained in endothelial cell media (R&D systems).

### Endothelial cell characterization

A total of 50,000 cells were plated per well of an 8-well glass chamber slide or a 24-well plate. Endothelial cell media (R&D) were changed every other day until cells reached the desired confluency. Media were aspirated and cells were fixed with 4% PFA for 10 min at room temperature. Cells were blocked in 5% normal goat serum (NGS), 3% bovine serum albumin (BSA), and 0.05% Triton X-100 for 1 h at room temperature and incubated with anti-CD31 (Abcam ab32457), anti-CA4 (R&D Systems MAB21861), or anti-RGCC (Developmental Studies Hybridoma Bank PCRP-RGCC-2C4) in blocking buffer overnight at 4 °C. Cells were washed 3 times with PBS and incubated with donkey anti-mouse 546 (Invitrogen A10036) or donkey anti-rabbit 546 (Invitrogen A10040) for 2 h at room temperature, washed 3 times, and counterstained with DAPI. Cells in 24-well plates were analyzed using epifluorescence (EVOS FL; AMG Life Technology). Cells grown on chamber slides were analyzed using confocal microscopy.

#### Acetylated LDL uptake

A total of 50,000 ECs were plated per well of a 24-well Matrigel-coated culture dish. Twenty-four hours later, 10 μg/ml of Alexa 488-labeled acetylated LDL (Thermo Fisher Scientific L233380) was added per well. ECs were incubated with acetylated LDL for 2–4 h. LDL uptake was analyzed using epifluorescence (EVOS FL; AMG Life Technology).

#### Tube formation

Wells of a 24-well plate were coated with 290 μl of Matrigel (10 mg/ml) for 1 h at 37 °C. iPSC-derived endothelial cells were dissociated with TryPLE, counted and plated at 100,000 cells per well in 300 μl of endothelial cell media. Transmission images were captured 1, 3, and 19 h post-seeding. At 21 h post-seeding, cells were washed 2 times in HBSS (+CA/+MG) and 8 μg/ml of calcein was added per well. Cells were imaged 1 h later at 22 h using epifluorescence (EVOS FL; AMG Life Technology).

#### Electron microscopy

Cells seeded in Matrigel for tube forming assays were fixed in one half strength Karnovsky fixative for at least 24 h prior to post-fixation with osmium tetraoxide (1% in 100 mM sodium cacodylate buffer, pH 7.2). After three 20-min washes in cacodylate buffer, cells were post-stained in 2.5% uranyl acetate in cacodylate buffer and dehydrated through graded ethanol (25–100%) prior to infiltration and embedment in Spurr’s low viscosity embedding media (Electron Microscopy Sciences Cat. No. 14300). All processing steps were conducted on the tissue culture plate. After curing the resin overnight at 70 °C, resin containing cells and Matrigel was removed from the culture dishes, trimmed, and sections collected on Formvar-coated grids at a thickness of approximately 85 nm. Images were collected on a transmission electron microscope (JEOL JEM1230).

#### Preparation of human choroid extracellular matrix scaffolds

Human donor punches were decellularized following our previously published protocol [25]. Human donor eyes were obtained from the Iowa Lions Eye Bank (Iowa City, IA), and all experiments were performed in accordance with the Declaration of Helsinki. In brief, 6-mm RPE/choroid punches from healthy individuals were decellularized as follows: Punches were transferred to a 24-well plate containing dH_2_O and incubated at room temperature for 1.5 h, dH_2_O was removed and replaced with 1% Triton X-100 for 3 h, and punches were washed 3 times for 5 min each in PBS followed by an overnight 18 h incubation in 1% Triton X-100 at 4 °C. Punches were washed with PBS 3 times, and 0.1% SDS+ 0.1 M EDTA was applied for 3 h at room temperature. Punches were washed 3 times with PBS and incubated with DNase I solution for 1 h at 37 °C followed by 3 washes in PBS and a final rinse for 1.5 h in dH_2_O.

#### Recellularization

Decellularized choroid punches were adhered to the bottom of a 24-well culture plate and held down by a drop of vacuum grease around the punches’ edge. iPSC-derived CECs were passaged using TryPLE, counted and plated at a density of 1 million cells in a 50-μl drop of media that was placed directly on top of the punch. Cells were allowed to settle on the punch for 30 min prior to the addition of an additional 450 μl of EC media. Cell culture media were replenished every 24 h. Four days after plating, punches were fixed in 4% paraformaldehyde. Punches were embedded in OCT-sucrose mixture. Seven-micrometer-thick cryosections were cut from a recellularized and control block. Immunofluorescence was performed for collagen IV (Abcam ab6586) as previously described [25]. All sections were counterstained with DAPI and mounted using Aqua-Mount. Images were captured on a BX-41 Olympus microscope at × 40 with a SPOT-RT camera.

## Results

### Differentiation of iPSC-derived choroidal endothelial cells

**Fig. 2:**
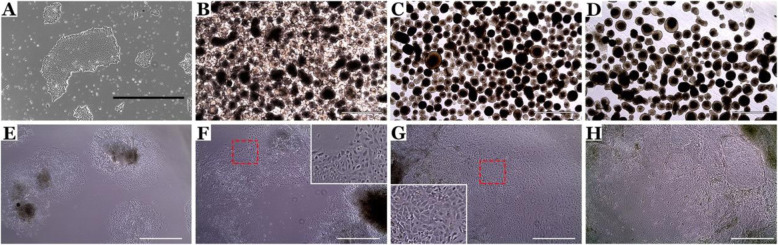
Differentiation of induced pluripotent stem cells into choroidal endothelial cells. Representative light microscope images acquired during the stepwise differentiation of induced pluripotent stem cells into mesoderm-derived choroidal endothelial cells. (**a**) iPSC colonies are expanded prior to differentiation and embryoid body (EB) formation. EBs were kept in suspension for 5 days. EB appearance after 1 (**b**), 3 (**c**), and 5 days (**d**) post-formation. On day 5, EBs were adhered to a Matrigel-coated 6-well plate at a density of 30 EBs per well. After 24 h (i.e., differentiation day 6), EBs have stuck to the Matrigel-coated surface (**e**). EBs gave rise to a heterogenous population of cells over 14 days. Areas of EC with cobblestone morphology can be seen at days 7 (**f**), 12 (**g**), and 14 (**h**). Note that red square in (**f**) and (**g**) demarcates the area depicted in the corresponding high-magnification inlay. Scale bars = 1000 μM

**Fig. 3:**
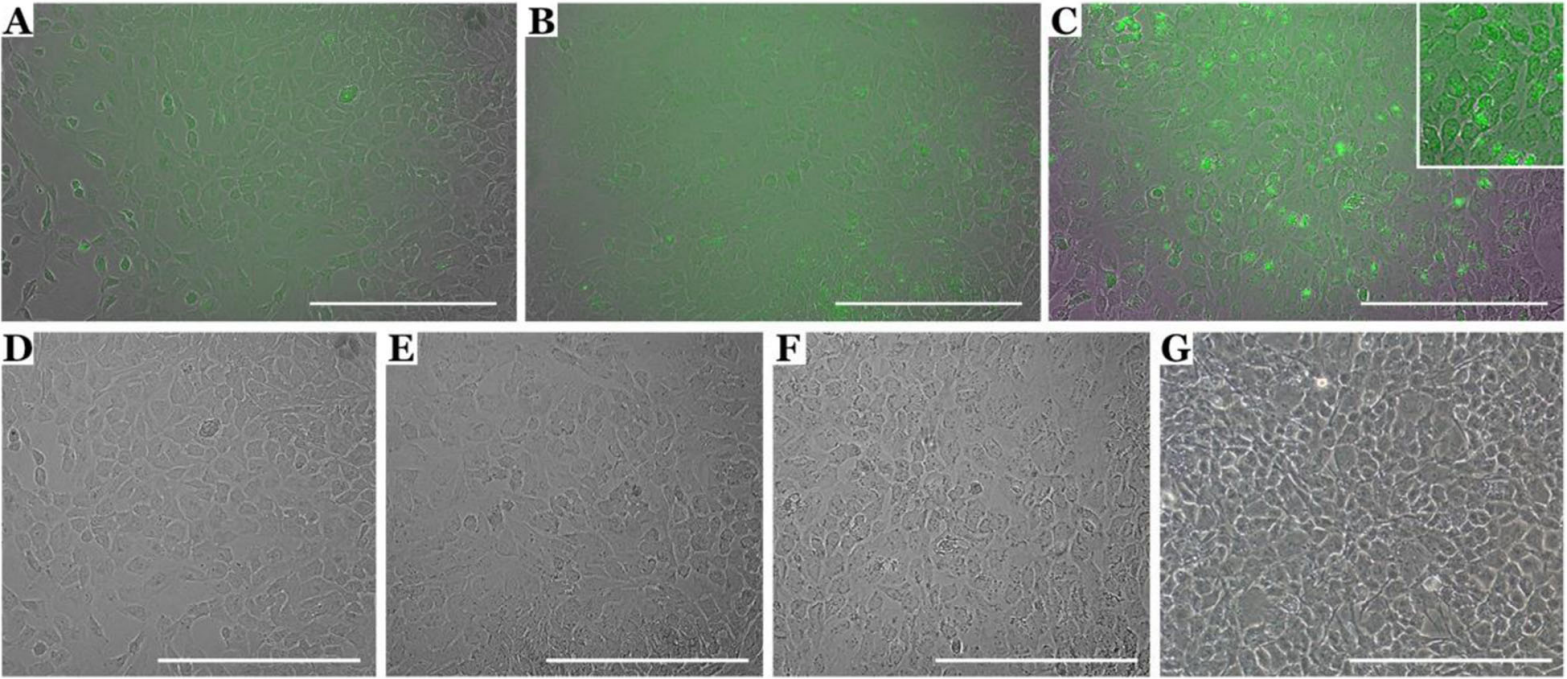
CDH5-GFP-positive cells are observed at day 11 of differentiation. (**a**) CDH5-GFP expressing cells are observed on day 11 (**a**, **d**), day 12 (**b**, **e**), and day 13 (**c**, **f**) during stepwise differentiation accompanied by a characteristic cobblestone morphology (**d–f**) which closely resembles choroidal endothelial cells immortalized from a healthy human donor eye (**g**). Scale bars = 400 μM. Note that inlay in (**c**) is a high-magnification image taken from the same field

We adopted previous publications, which use BMP-4, activin A, FGF, and VEGF to enrich for CD31-positive endothelial cells [20], and modified by using a stepwise transition from mTESR™1 to endothelial cell media with the addition of CTGF to specify choroidal endothelial cells [23]. HiPSCs were maintained on Matrigel-coated plates in mTESR™1 (Fig. 2a). Colonies were gently lifted enzymatically using dispase at 80% confluency ensuring colonies were kept intact (day 0). Colonies were allowed to settle by gravity and transferred to a low bind cell culture dish to prevent adherence and promote spherical embryoid body formation (Fig. 2b–d). BMP-4, activin A, and FGF2 were added on days 1, 2, and 3, respectively, to enrich EBs for mesoderm. On day 5, EBs were collected and allowed to settle onto a MG-coated 6-well plate at a density of 30 EBs per well supplemented with VEGF and CTGF. VEGF was included to promote endothelial cell differentiation, and CTGF was included to promote specification of CA4-positive choroidal endothelial cells [23]. On day 6, EBs could be observed adhering to the 6-well culture dish (Fig. 2e). Over 14 days, a mixed population of cells expand from the EBs with areas of cobblestone cuboidal cells (Fig. 2f–h) characteristic of ECs. When observed under epifluorescence, populations of endothelial cells positive for GFP (driven by the EC specific promoter CDH5) were observed on days 11, 12, and 13 (Fig. 3a–f) of differentiation which morphologically resemble the cobblestone morphology of HUVECS, and an immortalized choroidal endothelial cell line isolated from a human donor eye (Fig. 3g). On day 14, the heterogenous cell population was placed under zeocin selection to isolate a CDH5-enriched population. Alternatively, the cell monolayer was enzymatically dissociated using TryPLE and CD31-positive endothelial cells were isolated with anti-CD31 MACS beads. The zeocin selection and CD31 bead enrichment protocols were compared in subsequent immunocytochemical and functional assays.

### Characterization of ECs

**Fig. 4:**
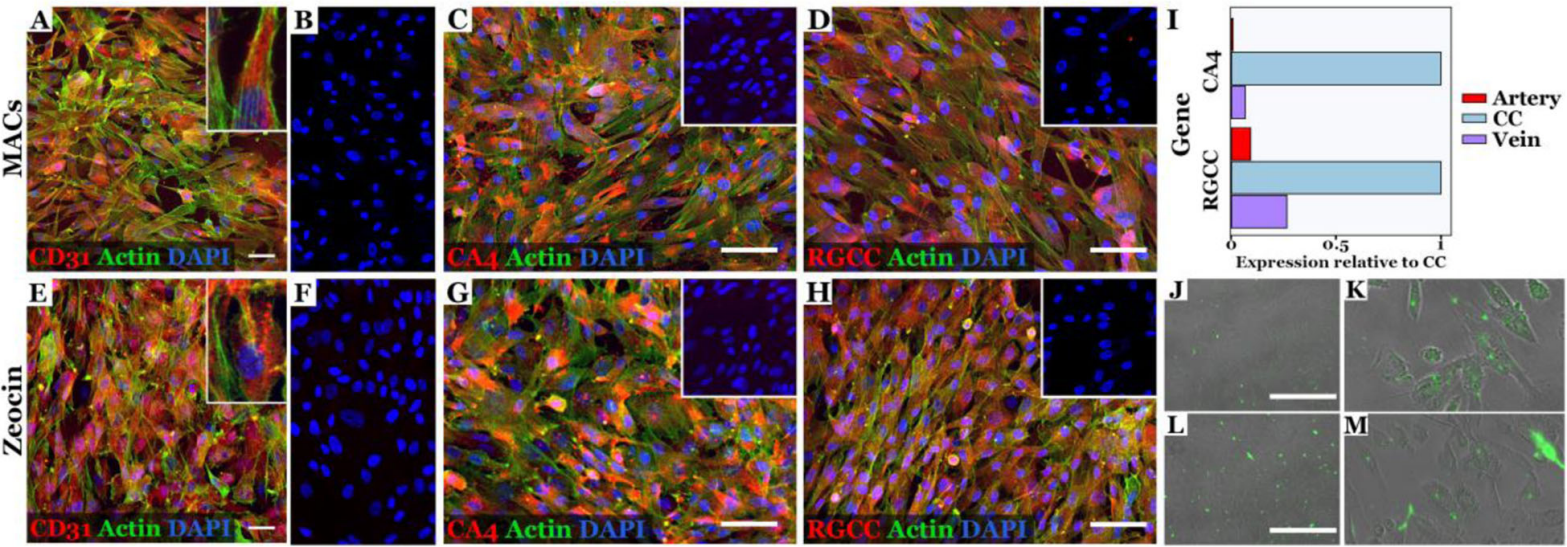
iPSC-derived choroidal endothelial cells express CD31, CA4, and RGCC. Representative immunocytochemical staining of endothelial cell-specific markers CD31 (**a**, **e** red), CA4 (**c**, **g** red), and RGCC (**d**, **h** red) in differentiated cells post-endothelial cell enrichment via CD31-positive MACS sorting (**a–d**) or CDH5 lentiviral zeocin selection (**e–h**). Positivity was determined via comparison to secondary antibody-only controls (**b**, **f** and inserts in top right corner). Cells were co-stained for actin (green), and nuclei were counterstained with DAPI. Scale bars = 50 μM. (**i**) CA4 and RGCC expression are enriched in primary human choriocapillaris endothelial cells (CC) opposed to primary human arterial (Artery) and venous (Vein) choroidal endothelial cells [26]. To calculate the relative expression of each gene compared to the choriocapillaris cluster (*x*-axis), the average expression of CA4 and RGCC were calculated for each cell type and divided by the mean expression in the choriocapillaris cluster. Alexa Fluro 488-labeled acetylated LDL uptake in iPSC-derived choroidal endothelial cells post-CD31 MACS sort (**j**, **k**) and zeocin selection (**l**, **m**)

iPSC-derived ECs labeled positively for the classical endothelial cell marker CD31 (Fig. 4a, e and Additional file 2) following expansion, and we observed 96.74% positivity post-MACS sort and 95.80% positivity post-zeocin selection (Table 1). Furthermore, our iPSC-derived ECs express the markers CA4 (Fig. 4c, g and Additional file 2) and RGCC (Fig. 4d, h and Additional file 2), which, as compared to endothelial cells lining arteries and veins, are both highly abundant in choriocapillaris endothelial cells (Fig. 4i). 88.90% of cells stained positively for CA4 post-MACS sort and 88.98% stained positively post-zeocin selection while 95.01% of cells were positive for RGCC following MACS sort and 95.78% were positive following zeocin selection. We compared iPSC-derived CECs to a primary immortalized human donor CEC line and observed similar staining patterns for CD31, CA4, and RGCC using epifluorescence microscopy (Additional file 3).

**Table 1:** Percent of cells expressing each marker following selection

	Marker	Percent Positive C1	Percent Positive C2	Percent Positive C3	Mean positive
MACS	CD31	95.93	97.73	96.55	96.74
	CA4	90.97	88.89	86.84	88.90
	RGCC	96.99	96.88	91.18	95.01
Zeo	CD31	96.84	96.55	94.00	95.80
	CA4	87.94	87.50	91.49	88.98
	RGCC	97.18	95.16	95.00	95.78

### Characterization of EC function

**Fig. 5:**
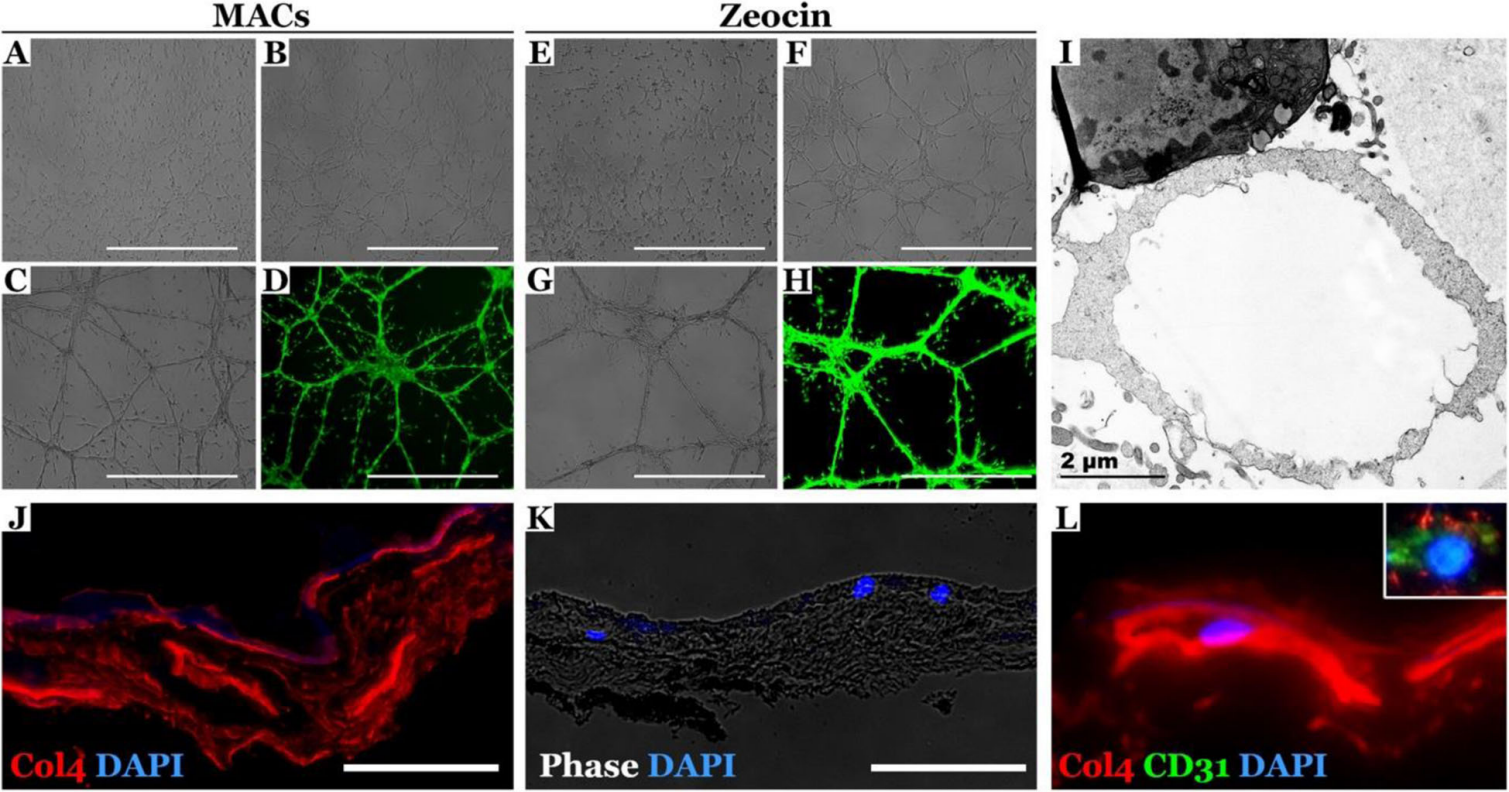
iPSC-derived choroidal endothelial cells form vascular tubes in Matrigel and can migrate into decellularized human choroid extracellular matrix. Microscopy images demonstrate formation of vasculature 1 (**a**, **e**), 3 (**b**, **f**), 19 (**c**, **g**), and 22 h (**d**, **h**) post-seeding of iPSC-derived CECs into a 3D Matrigel matrix (**a–d**): CD31 MACS sorted cells; (**e–h**): zeocin selected cells. Calcein was used at the 22-h time point (**d**, **h**) to demonstrate cell viability. Scale bars = 1000 μM. (**i**) Transmission electron micrograph of iPSC-derived CEC tube demonstrates lumen formation. (**j**) Immunofluorescence labeling of human RPE/choroid after decellularization. Anti-collagen IV (red) highlights the structure of the choroid tissue following decellularization, and the absence of nuclear DAPI (blue) demonstrates the absence of native endothelial cells. iPSC-derived CECs are observed within the vascular tubes of the choriocapillaris (**k**, **l**) in both a brightfield and anti-collagen IV labeled image

The internalization of acetylated low-density lipoprotein (ac-LDL) and Matrigel tube formation assays are commonly used to assess EC function in vitro. In addition to expression of specific cell surface markers, iPSC-derived ECs were able to uptake ac-LDL (Fig. 4j–m) and form tubes similar to primary human choroidal ECs in Matrigel (Additional file 3) following either MACS (Fig. 5a–d) or zeocin selection (Fig. 5e–h). Electron microscopy of the tubes indicated the tubes contain a lumen (Fig. 5i), suggesting that these cells have the ability to participate in angiogenesis. Decellularized human donor choroidal extracellular matrix allows us to test the ability of iPSC-derived CECs to migrate into and repopulate empty choroidal vessels. We observed multiple CECs in empty choroidal vessels (Fig. 5, note presence of DAPI (k, l) and expression of CD31 (l; high mag inlay) in donor iPSC-CECs) compared to a control punch in which no iPSCs were added (Fig. 5j, lack of DAPI-positive native cells).

## Discussion

iPSC technology allows us to generate endothelial cells from patient fibroblasts, providing a valuable resource for studying patient-specific ECs in vitro. In addition, patient-derived ECs could be a valuable resource for cell replacement to repair damaged vessels. Successful cell replacement strategies are organ specific and likely require the transplantation of ECs that closely resemble those that are lost in disease. In the eye, the ECs which line the choriocapillaris are unique in their structure and function; therefore, to accurately model retinal degenerative diseases such as AMD, we require a protocol to reliably produce CECs from iPSCs.

Our group previously published a method to produce murine CECs via co-culture [27], and furthermore, using RNA-Seq identified CTGF as a key factor promoting CEC-like differentiation in human iPSCs [23]. In our previous study, we employed spontaneous differentiation of EBs to generate mesoderm precursors, followed by the addition of CTGF to direct mesoderm differentiation toward CECs. Early endothelial cell protocols utilized spontaneous differentiation of EBs to obtain ECs [18]. However, significant increases in EC yield were achieved by using a directed approach. This involves the addition of growth factors important in specifying mesoderm during embryonic development, including BMP-4 and FGF, which derive from the epiblast, and activin A, which derives from the hypoblast [19, 20]. Adding these factors during EB formation can steer germ layer differentiation toward a mesoderm cell fate, which in turn leads to increased yield of EC differentiation. In order to improve CEC differentiation to ensure reproducible results in iPSCs derived from multiple donors, we adopted a directed approach. Prior to differentiation, we transduced 3 iPSC donor lines with a lentiviral reporter. This lentivirus contains CDH5 driving GFP with zeocin resistance, allowing us to track EC differentiation, while also providing a unique selection method for iPSC-derived ECs. We opted to use EBs for our first step of differentiation and slowly transitioned aggregating iPSC colonies from mTESR™1 to endothelial cell medium with stepwise addition of BMP-4, activin A, and FGF-2 (Fig. 1). On day 5, EBs were adhered to a Matrigel-coated plate. We added VEGF and CTGF to promote differentiation of choroidal endothelial cells. Previously, we have reported that CTGF is sufficient to drive CECs [23]. CTGF is a matricellular protein that is important for vessel growth during retinal development [28] and has been shown to promote endothelial cell proliferation, migration, and tube formation in vitro [29]. On day 11, we observed GFP-positive subpopulations of cells, indicating a commitment to an endothelial cell fate. On day 14, we observed a heterogeneous population of cell types. In order to purify endothelial cells, we adopted two independent positive selection methods. The first takes advantage of the zeocin selection cassette in our reporter lentivirus. Placing cells under zeocin selection for 48 h, we saw drastic cell death of non-CDH5-positive cells. Alternatively, we tested whether we could purify endothelial cells using a non-lentiviral method. We choose to use CD31-positive MACS beads to magnetically separate CD31-expressing endothelial cells from other cell types. We observed that approximately 7% of overall cells were CD31 positive.

Following both selection methods, we expanded the endothelial cells and used immunohistochemistry to verify expression of CD31. Regardless of selection method, we obtained pure populations of cells expressing this classical endothelial cell marker. Although both selection methods gave rise to cells that express endothelial cell markers, they do appear slightly different morphologically. Specifically, CD31 selected cells look more endothelial like, retaining a cobblestone morphology. In contrast, cells selected with zeocin appear more elongate and fibroblast like. Zeocin causes cell death by inducing double-stranded DNA breaks and is therefore harder on the cells. It is likely that only the most robust endothelial cells that are more proliferative survive zeocin selection.

As indicated above, choriocapillaris loss occurs early in the pathogenesis of AMD, often preceding degeneration of the overlying RPE and photoreceptor cells [8–10]. As such, autologous iPSC-derived CEC replacement early in the disease course could be advantageous in slowing AMD progression, including the atrophic dry form of the disease, which currently has no treatment. AMD patients with a polymorphism in the *CFH* gene (Y402H) have increased risk of CEC loss [30]. In order to restore healthy CECs, iPSC CRISPR technology could be used to restore wildtype CFH, and these CRISPR-corrected iPSCs would then be differentiated to CECs to be used for cell transplantation. For such a strategy, the best approach for deriving iPSC-CECs would be to utilize the CD31 MACS selection method, as it does not require lentiviral integration and produces cells which are morphologically superior.

In order to determine whether our EC population reassembled ECs in the choriocapillaris, we immunolabeled for carbonic anhydrase IV (CA4), a specific marker for ECs of the choroid capillaries. CA4 is visible immunohistochemically during development of the choroidal vasculature [31] and is observed in the choriocapillaris but not in the larger vessels in Sattler’s or Haller’s layers in adulthood [32]. Our differentiation protocol produced ECs positive for CA4, suggesting successful reassembly of ECs in the choriocapillaris. Recently, our group has identified an additional marker, regulator of cell cycle gene (RGCC), that is useful for distinguishing endothelial cells in the choriocapillaris from ECs lining arteries or veins [26]. Like CA4, RGCC is more abundant in choroid capillary ECs than other ECs in the choroid. In addition to CA4 positivity, we found that our iPSC-ECs stain positively for RGCC providing strong evidence that we obtained choriocapillaris-specific ECs.

A hallmark of ECs is the ability to form capillary tube-like networks. Here, we show that iPSC-derived ECs organized into 3-dimensional tube networks in Matrigel. Using electron microscopy, we confirm the presence of vascular lumens within the tubular network. To determine whether iPSC-derived CECs could be used for cell replacement strategies, we tested their ability to migrate into and repopulate empty choriocapillaris vessels. Decellularization of human donor RPE/choroid creates an empty ECM scaffold [25]. We observed that iPSC-derived CECs can revascularize the empty choriocapillaris tubes. This will begin to allow us to develop cell replacement strategies for age-related macular degeneration, which has a hallmark feature of early choroidal endothelial cell loss. Thus, we have developed a stepwise protocol to successfully generate iPSC-derived CECs that morphologically and structurally resemble ECs of the choriocapillaris.

## Conclusions

In summary, we have developed a stepwise differentiation protocol to generate patient-specific choroidal endothelial cells. We have demonstrated two methods to purify CECs post-differentiation, making this protocol translatable to multiple laboratory settings. We have provided a tool to successfully study CECs in vitro for disease modeling in AMD and other choroid-related ocular diseases.

## Supplementary information

**Additional file 1.** Detailed list of reagents, equipment and step by step protocol for differentiation of choroidal endothelial cells.

**Additional file 2.** iPSC-derived choroidal endothelial cells express CD31, CA4 and RGCC.

**Additional file 3.** Comparison between iPSC-derived choroidal endothelial cells and a primary human choroidal endothelial cell line for expression of CD31, CA4 and RGCC.

**Additional file 4.** Human primary choroidal endothelial cell line is capable of forming tubes in a 3D Matrigel matrix.

## Data Availability

The datasets supporting the conclusions of this article are included within the article and its additional files.

## Abbreviations

ECs: Endothelial cells
CEC: Choroidal endothelial cell
AMD: Age-related macular degeneration
iPSCs: Induced pluripotent stem cells
CTGF: Connective tissue growth factor
RPE: Retinal pigment epithelium
RGCC: Regulator of cell cycle
CA4: Carbonic anhydrase IV
