# Supplementary material for: Stepwise differentiation and functional characterization of human induced pluripotent stem cell-derived choroidal endothelial cells
Source: Stem Cell Res Ther. 2020 Sep 23;11:409. doi: 10.1186/s13287-020-01903-4 (PMC7510078; doi:10.1186/s13287-020-01903-4)
Supplement: Supplementary file 1 — Additional file 1. Detailed list of reagents, equipment and step by step protocol for differentiation of choroidal endothelial cells. [file 13287_2020_1903_MOESM1_ESM.docx]

**Materials and Equipment**

- mTESR^TM^1 (Cat. No. 85850; STEMCELL Technologies, USA)
- Dispase (Cat. No. 17105041; ThermoFisher Scientific)
- Matrigel (Cat. No. 354234; Corning)
- DMEM (Cat. No. 11965-092; ThermoFisher Scientific)
- Primocin^TM^ (Cat. No. ant-pm-1; Invivogen, San Diego, CA, USA) *optional*
- Blasticidin S HCL (Cat. No. A1113902; ThermoFisher Scientific)
- Endothelial cell growth media (Cat. No. CCM027; R&D systems, Minneapolis, USA)
- 6 well culture plates (Cat. No. 3516; Corning)
- 60 mm petri dish (Cat. No. 1007; Corning)
- PolyHema (Cat. No. P3932; Sigma-Aldrich) *optional replacement 60 mm ultra-low attachment culture dish (Cat. No. 3261 Corning)
- Activin A (Cat. No. 338-AC-010; R&D systems)
- BMP4 (Cat. No. 130111168; Miltenyi Biotec)
- FGF-2 (Cat. no. 338-AC-010; R&D systems)
- VEGFa (Cat. No. 100-20; Peprotech)
- CTGF (Cat. No. 120-19; Peprotech)
- Zeocin (Cat. No. R25001; ThermoFisher Scientific)
- CD31 MACS beads (Cat. No. 130091935; Miltenyi Biotec)
- TrypLE Express Enzyme 1x (Cat. No. 12604013; ThermoFisher Scientific)
- 0.04% non-acetylated BSA (Cat. No. B90000; New England Biolabs)
- Dulbecco’s Phosphate Buffered Saline (Cat. No. 15040066; ThermoFisher Scientific)
- 10 ml stripette (Cat. No. 4488; Corning)
- 15 ml sterile conical tubes (Cat. No. 229411; CELLTREAT)
- CDH5-AbR-GFP (Cat. No. 122970; Addgene)
- Automated cell counter or hemocytometer
- 37°C, 5% CO_2_ incubator
- Miltenyi autoMACS Pro Separator

**Lentiviral production**

CDH5p-GFP-ZEO lentiviral vector was constructed incrementally. First, the CDH5 promoter was cloned into a pENTR5’-TOPO vector. Then, GFP-T2A-Zeocin was amplified from pGreenZEO (System Biosciences; Cat. No. SR500VA/PA) and subcloned into pENTR/D-TOPO. Finally, the pENTR5’CDH5p and pENTR/D-GFP-ZEO were cloned into the pDEST R4R3 Vector II (Thermo Fisher Scientific; Cat. No. A11145) using Gateway™ LR Clonase™ II Enzyme mix (Thermo Fisher Scientific; Cat. No. 11791100). Note that the pDEST R4R3 Vector II includes a blasticidin resistance cassette for selection. The resulting vector was submitted to the Viral Vector Core Facility at the University of Iowa for lentiviral packaging. The lentiviral plasmid generated using this approach has been deposit with Addgene (Cat # 122970).

**Expansion of iPSCs for viral transduction**

1. Expand hiPSCs in mTeSR^TM^1 base media containing 5x supplement and primocin (100 ug/mL) on Matrigel coated six well tissue culture plates.

*Note: Matrigel stock concentrations vary between 5-10 mg/mL as specified by the manufacturer. Thaw Matrigel overnight at 4°C and make aliqouts using eppendorfs, pipette tips and a rack pre-cooled at -20°C. Aliquot the required amount for 150 ug in 50 ml (3 ug/ml in 50 ml) based on the stock concentration. Work quickly as Matrigel solidifies and forms a gel when warmed. Store aliquots at -20°C and resuspend in 50 ml of cold DMEM. 2 ml of MG in DMEM is used per well of a 6 well culture plate. Plates should be incubated for at least 30 minutes at 37°C.*

**Viral transduction of iPSCs**

**Day 1**

1. Aspirate media from iPSCs and add 1 ml of dispase per well. Incubate at 37°C for 4-8 minutes.
2. Aspirate dispase and gently wash once with DMEM.
3. Aspirate DMEM and use 1 ml per well of fresh mTeSR^TM^1 to dislodge cells with a 1 ml pipette. Transfer cells to a 15 ml conical tube.
4. Centrifuge cells for 3 minutes at 700g.
5. Gently aspirate media and resuspend cell pellet in 1 ml of fresh mTeSR^TM^1.
6. Count cells using an automated cell counter and plate at a density of 50,000 cells per well of a 24 well plate. Cells will attach within 24 hours.

**Day 2**

1. Aspirate media and replace with 500 ul of fresh mTeSR^TM^1.
2. Add lentiviral particles for a final multiplicity of infection (MOI) of 10 (i.e. 10 viral particles per cell).

*Note: Include an untransduced control i.e.: a well of iPSC that did not receive lentiviral particles as a control.*

**Day 4**

1. Aspirate media containing lentiviral particles and replace with mTeSR^TM^1 containing blasticidin (5 ug/ml).

**Day 5**

1. Maintain cells in mTeSR^TM^1 containing blasticidin (5 ug/ml) for 5-7 days changing medium daily.

**Differentiation of iPSC derived choroidal endothelial cells**

**Day 0**

Embryoid Body (EB) formation

*Note: One day prior to EB formation 60 mm cell culture plates should be pre-coated with polyHEMA. Add 2 ml of poly-HEMA per plate and swirl to ensure entire surface is coated. Remove excess and allow to airdry over night with the lid off in the culture hood. PolyHEMA prevents iPSC colony adhesion. Optional: Ultralow adhesion plate.*

1. When iPSC colonies reach 80% confluency aspirate mTESR ^TM^1 from 3 wells of a 6 well cell culture dish. Add 1 ml of dispase per well for 4-8 minutes or until the edges of the iPSC colonies begin to curl.

*Note: Other wells of the 6 well plate can be used for cryopreservation and continued culture.*

1. Aspirate dispase and wash gently with DMEM.
2. Remove DMEM and use a 10 ml stripette to add 1 ml of fresh DMEM per well and gently lift colonies as to prevent colonies from breaking. Repeat this step twice to insure maximal recovery of intact colonies. Transfer colonies to a 15 ml conical tube.
3. Allow colonies to settle by gravity for 5-10 minutes.
4. Remove excess DMEM from settled colonies and resuspend in 6 ml mTESR ^TM^1 and 3 ml endothelial cell growth media. Transfer the colonies gently with a 10 ml pipette into a 60 mm culture dish precoated with polyHEMA.

**Day 1**

1. Using a 10 ml stripette gently transfer EBs to a 15 ml conical tube and allowed to settle by gravity.
2. Carefully aspirate media with a stripette.
3. Resuspend EBs in 3 ml of mTeSR^TM^1 and 6 ml endothelial cell media supplemented with 20 ng/ml BMP-4 and transfer back into the 60 mm culture dish.

**Day 2**

1. Using a 10 ml stripette gently transfer EBs to a 15 ml conical tube and allow to settle by gravity.
2. Carefully aspirate media with a stripette.
3. Resuspend EBs in 9 ml of endothelial cell media supplemented with 20 ng/ml BMP-4 and 10 ng/ml activin A and transfer back into the 60 mm culture dish.

**Day 3**

1. Using a 10 ml stripette gently transfer EBs to a 15 ml conical tube and allow to settle by gravity.
2. Carefully aspirate media with a stripette.
3. Resuspend EBs in 9 ml endothelial cell media supplemented with 20 ng/ml BMP-4, 10 ng/ml activin A and 8 ng/ml FGF-2 and transfer back into the 60 mm culture dish.

EB adherence

**Day 5**

1. Precoat 3 wells of a 6-well plate with Matrigel at 37°C for at least 30 minutes.
2. Collect EBs by gently pipetting all 9 ml of media from the 60 mm culture dish and transfer to a 15 ml conical tube.
3. Allow EBs to settle by gravity.
4. Aspirate Matrigel from 6-well plate and replace with 2 ml of endothelial cell media supplemented with 10 ng/ml BMP4, 8 ng/ml FGF-2, 25 ng/ml VEGFa and 25 ng/ml CTGF.
5. Remove as much media as possible from the EBs and resuspend in 2 ml of endothelial cell media supplemented with 10 ng/ml BMP4, 8 ng/ml FGF-2, 25 ng/ml VEGFa and 25 ng/ml CTGF.
6. Using a 10 ml stripette transfer 30 EBs per well of the Matrigel coated 6-well plate.

**Day 6**

1. Aspirate media from 6-well plate and add 2 ml per well of endothelial cell media supplemented with 10 ng/ml BMP4, 8 ng/ml FGF-2, 25 ng/ml VEGFa and 25 ng/ml CTGF.

**Day 8**

1. Aspirate media from the 6-well plate and add 2 ml per well of endothelial cell media supplemented with 8 ng/ml FGF-2, 25 ng/ml VEGFa and 25 ng/ml CTGF.

**Day 9-14**

1. Maintain cells in endothelial cell media supplemented with 8 ng/ml FGF-2, 25 ng/ml VEGFa and 25 ng/ml CTGF replacing media every other day.

**Day 14**

Selection for endothelial cell population

1. Zeocin selection
2. Aspirate media and replace with endothelial cell media containing 25 ng/ml of zeocin for 48 hours.
3. Following 48 hours of selection, CECs can be maintained in complete CEC media until harvest or passage.
4. Following selection cells can be passaged for at least 3 passages.

OR

1. CD31 Microbead MACS sort
2. Aspirate media and add 1 ml of TryPLE per well. Incubate for 5 minutes or until cells start to round up into single cells.
3. Add 1 ml of media to each well and collect cell suspension in a 15 ml conical tube.
4. Centrifuge cells at 600g for 5 minutes.
5. Gently aspirate media and resuspend pellet in PBS to a final concentration of 1x10^7 cell/ml.
6. Add 20 ul of FCR blocking agent per 60 ul of cells as per manufacturer instructions.
7. Add 20 ul of CD31 beads per 60 ul of cells and incubate at 4°C for 15 minutes with gentle shaking.
8. Add 1 ml of PBS and centrifuge at 500 g for 5 minutes.
9. Aspirate supernatant and resuspend pellet in 1 ml of PBS containing 0.04% non-acetylated BSA.
10. Sort cells on an autoMACs sorter using the selection program “possel”.
11. Centrifuge the positive fraction at 600 g for 5 minutes.
12. Aspirate buffer and resuspend in 2 ml of endothelial cell media.
13. Plate cells into one well of a 6 well plate pre-coated with Matrigel.

*Note. Approximately 7% of sorted cells should be in the positive fraction.*

1. Following selection cells can be passaged for at least 3 passages.
