# Supplementary material for: Stepwise differentiation and functional characterization of human induced pluripotent stem cell-derived choroidal endothelial cells
Source: Stem Cell Res Ther. 2020 Sep 23;11:409. doi: 10.1186/s13287-020-01903-4 (PMC7510078; doi:10.1186/s13287-020-01903-4)
Supplement: Supplementary file 2 — Additional file 2. iPSC-derived choroidal endothelial cells express CD31, CA4 and RGCC. [file 13287_2020_1903_MOESM2_ESM.docx]

**
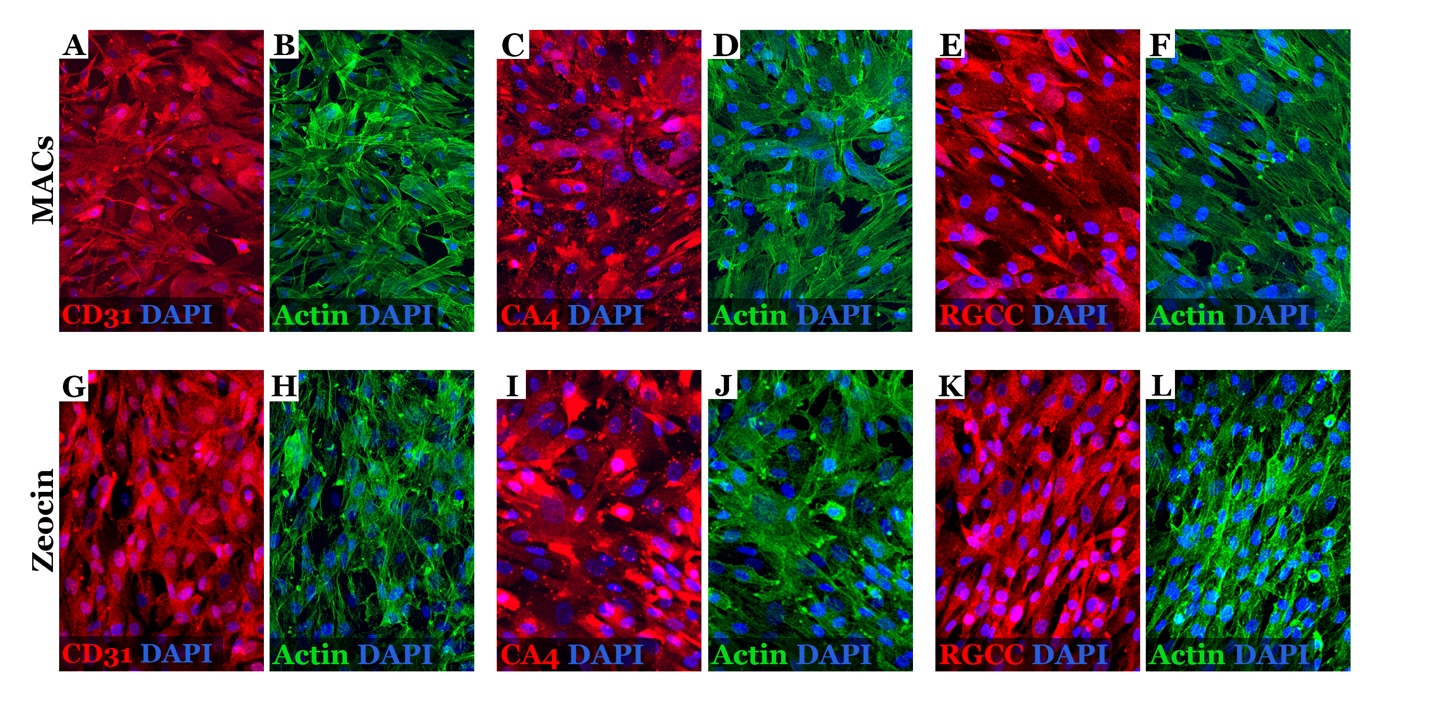
Additional file 2.** **iPSC-derived choroidal endothelial cells express CD31, CA4 and RGCC.** Representative immunocytochemical staining of endothelial cell specific markers CD31 (**A**, **G** red), CA4 (**C**, **I** red) and RGCC (**E, H** red) in differentiated cells post-endothelial cell enrichment via CD31 positive MACs sorting (**A-F**) or CDH5 lentiviral zeocin selection (**G-L**). Cells were co-stained for actin (B, D, F, H, J & L green) and Nuclei were counterstained with DAPI.
