# Supplementary material for: Stepwise differentiation and functional characterization of human induced pluripotent stem cell-derived choroidal endothelial cells
Source: Stem Cell Res Ther. 2020 Sep 23;11:409. doi: 10.1186/s13287-020-01903-4 (PMC7510078; doi:10.1186/s13287-020-01903-4)
Supplement: Supplementary file 3 — Additional file 3. Comparison between iPSC-derived choroidal endothelial cells and a primary human choroidal endothelial cell line for expression of CD31, CA4 and RGCC. [file 13287_2020_1903_MOESM3_ESM.docx]

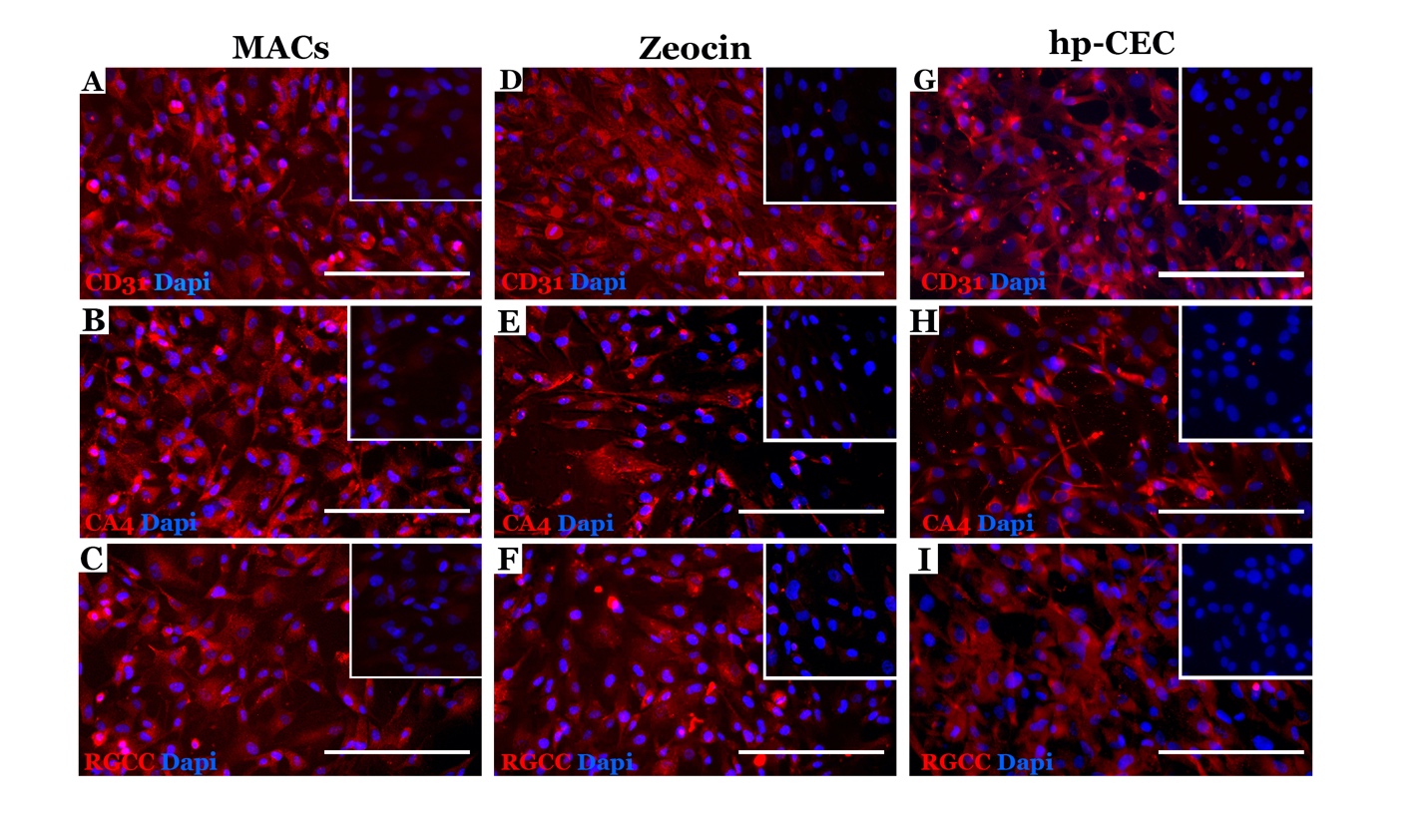


**Additional file 3.** **Comparison between iPSC-derived choroidal endothelial cells and a primary human choroidal endothelial cell line for expression of CD31, CA4 and RGCC.** Representative immunocytochemical staining of endothelial cell specific markers CD31 (**A, D & E,** red), CA4 (**B**, **E &** **H,** red) and RGCC (**C, F & H,** red) in iPSC-derived ECs post-CD31 MACs sorting (**A-C**), iPSC-derived ECs post-CDH5 lentiviral zeocin selection (**D-F**) and a human primary choroidal endothelial cell line (**G-I**). Scale bars = 200uM. Note that the pattern of staining between iPSC-derived choroidal endothelial cells is identical between iPSC-CECs and primary human CECs.
