# Supplementary material for: Stepwise differentiation and functional characterization of human induced pluripotent stem cell-derived choroidal endothelial cells
Source: Stem Cell Res Ther. 2020 Sep 23;11:409. doi: 10.1186/s13287-020-01903-4 (PMC7510078; doi:10.1186/s13287-020-01903-4)
Supplement: Supplementary file 4 — Additional file 4. Human primary choroidal endothelial cell line is capable of forming tubes in a 3D Matrigel matrix. [file 13287_2020_1903_MOESM4_ESM.docx]

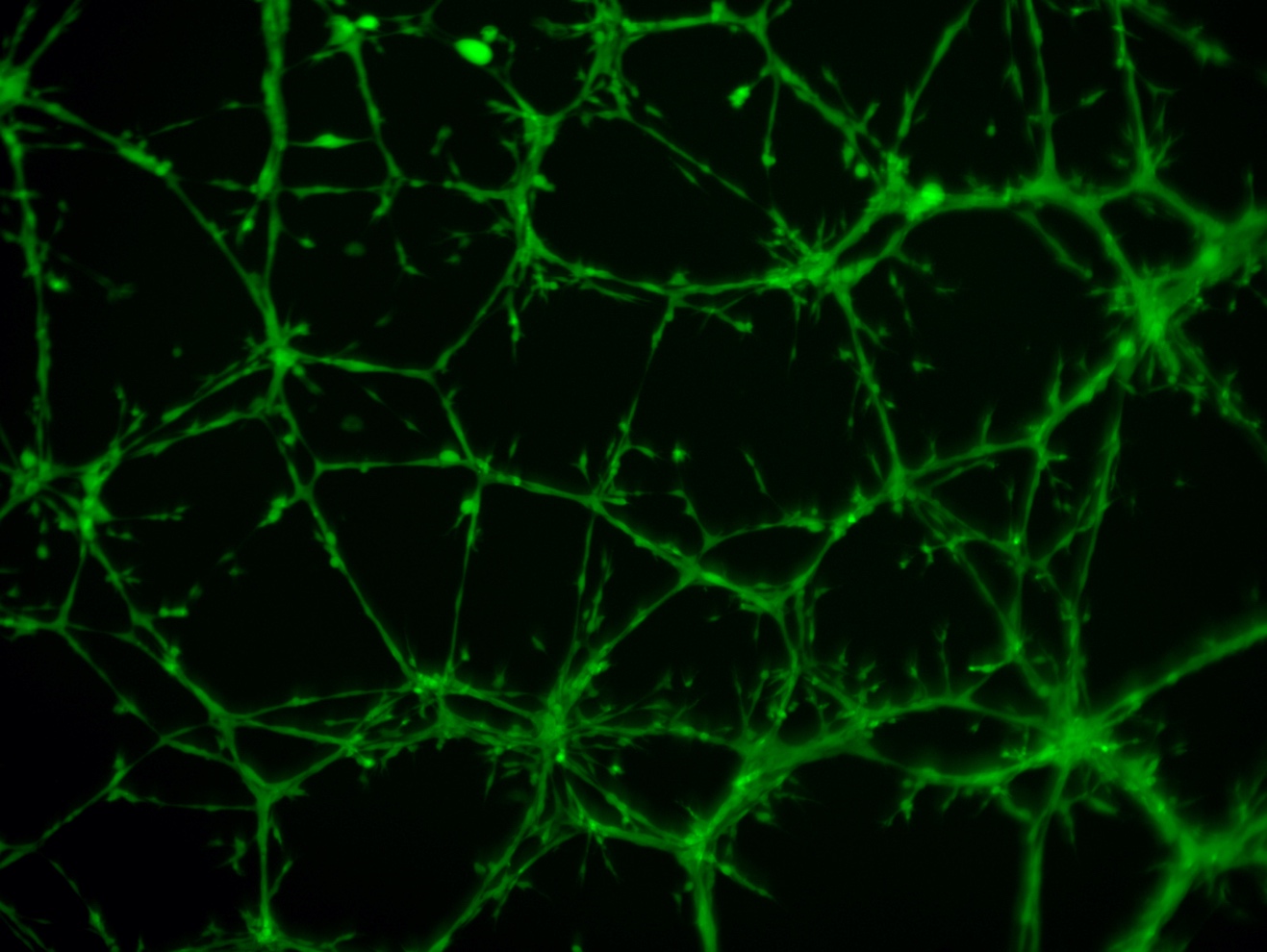


**Additional File 4.** Microscopy image demonstrating the ability of a human primary choroidal endothelial cell line to form vasculature tubes at 22-hours post-seeding into a 3D Matrigel matrix. Calcein (green) was used to demonstrate cell viability.
